# Flexibilities provided by the Agreement on Trade-Related Aspects of Intellectual Property Rights

**DOI:** 10.2471/BLT.17.206896

**Published:** 2018-03-01

**Authors:** Carlos M Correa

**Affiliations:** a Centre for Interdisciplinary Studies of Industrial Property and Economics, University of Buenos Aries, Avenida Figueroa Alcorta 2263, 1er.piso (1425) Buenos Aires, Argentina

In 1995, when the Agreement on Trade-Related Aspects of Intellectual Property Rights (TRIPS) came into effect, all but the least-developed members of the World Trade Organization (WTO)^1^ were bound to provide intellectual property protection for pharmaceutical products. The proponents of the Agreement had argued that the global strengthening of such protection would spur innovation. However, after the Agreement came into effect, there was a fall in the number of new medicines being approved annually. 

So far, the main impact of the Agreement on TRIPS has been on the prices of medicines. In the absence of competition, prices can be fixed – by the owners of the relevant intellectual property rights – on the basis of what the market can bear. Although treatment of hepatitis C with sofosbuvir can cost as much as 84 000 United States dollars (US$) per patient in countries where the drug has patent protection, it is expected to cost less than US$ 300 in countries where the product has not been covered by a valid patent.^2^

To minimize the problems caused by the Agreement, academics, governments of low-income countries, many nongovernmental organizations, the World Health Organization (WHO) and other United Nations organizations pay special attention to the Agreement’s so-called flexibilities.^3^^,^^4^ In this issue of the *Bulletin*, Ellen ‘t Hoen and others summarize the use of some of these flexibilities over 15 years.^5^ These flexibilities – e.g. compulsory licenses, government use for non-commercial purposes, non-exclusive protection of test data and parallel imports – can be used to mitigate the detrimental impact of the Agreement’s provisions on market dynamics and access to medicines.

The right of WTO members to exploit such flexibilities was confirmed at WTO’s Ministerial Conference in 2001.^3^ After 2015, both the United Nations Secretary-General’s High Level Panel on Access to Medicines^6^ and the panel that conducted a review of *WHO’s global strategy and plan of action on public health, innovation and intellectual property*^7^ highlighted the importance of the flexibilities in the Agreement on TRIPS. The extent to which such flexibilities have already been incorporated into national laws and practice shows substantial variation. Several compulsory licenses – allowing a company to produce a patented product or process without the consent of the patent owner – have been issued for medicines, mainly to treat infections with human immunodeficiency virus. Most of these licenses have led to substantial reductions in the costs of treatment. The use of such licenses is not limited to low- and middle-income countries. In Germany, for example, the Federal Court of Justice confirmed a compulsory license that allowed Merck to continue the commercialization of an antiretroviral drug for which another company, Shionogi, held a patent.^8^

Another important flexibility is provided by the definition of the standards of patentability, and the rigour with which they are applied in determining whether a claimed invention is patentable. Weaknesses or gaps in such standards can allow ever-greening by the pharmaceutical industry – e.g. the obtaining of additional patents on a different crystalline form, a new formulation or new use of a known medicine – which may be enforced to block or delay the market entry of generic equivalents. Although several countries have fine-tuned their patentability standards in order to limit ever-greening, many countries still apply inappropriately broad criteria.

Research has shown that the TRIPS flexibilities are poorly exploited and that much more could be done to align intellectual property protection with public health policies. Actions include adopting patentability guidelines, streamlining the procedures for granting compulsory licenses and training patent examiners and judges. One of the objectives of the 2018 Global Summit on Intellectual Property and Access to Medicine was to provide a strategic platform to discuss successes and setbacks for governments and civil society in resisting trade, lobbying and litigation threats that undermine the use of TRIPS flexibilities’.^9^ UNITAID’s call for proposals supporting access to medicines through innovative use of TRIPS flexibilities^10^ also points to the need for further action in this area.

In determining the extent to which the flexibilities are used, issues of classification may arise.^5^ The extent may be easily overestimated if, for example, we counted as a use of a TRIPS flexibility every patent application rejected due to a rigorous examination or every purchase of generic drugs made by the government of a low-income country in a situation where intellectual property rights could have been enforced to prevent generic substitutions. The data should show whether the use took place in low-, middle- or high-income countries, as in the use of compulsory licenses and other flexibilities the main challenges are faced by the latter. 

To support the more extensive exploitation of the flexibilities provided by the Agreement on TRIPS, we need a continuous effort from academics, governments, international and nongovernmental organizations. The health of a large part of the world’s population depends on timely and effective action.
